# Phenylalanyl-tRNA synthetase deficiency caused by biallelic variants in *FARSA* gene and literature review

**DOI:** 10.1186/s12920-023-01662-0

**Published:** 2023-10-13

**Authors:** Ruolan Guo, Yuanying Chen, Xuyun Hu, Zhan Qi, Jun Guo, Yuchuan Li, Chanjuan Hao

**Affiliations:** 1 Beijing Key Laboratory for Genetics of Birth Defects, MOE Key Laboratory of Major Diseases in Children, National Center for Children’s Health, Beijing Pediatric Research Institute, Beijing Children’s Hospital, Capital Medical University, Beijing, 100045 China; 2 Outpatient Department, National Center for Children’s Health, Beijing Children’s Hospital, Capital Medical University, Beijing, 100045 China

**Keywords:** Exome sequencing, *FARSA* gene, FARSA-deficiency

## Abstract

**Background:**

Aminoacyl-tRNA synthetases (ARSs) are indispensable enzymes for protein biosynthesis in cells. The phenylalanyl-tRNA synthetase (FARS1) located in cytoplasm which consists of two FARS alpha subunits (FARSA) and two FARS beta subunits (FARSB). Autosomal recessive inheritance of pathogenic variants of *FARSA* or *FARSB* can result in defective FARS1 which are characterized by interstitial lung disease, liver disease, brain abnormalities, facial dysmorphism and growth restriction.

**Methods:**

Exome sequencing was used to detect the candidate variants. The in silico prediction and expressional level analysis were performed to evaluate the pathogenicity of the variations. Additionally, we presented the patient’s detailed clinical information and compared the clinical feature with other previously reported patients with FARSA-deficiency.

**Results:**

We identified compound heterozygous rare missense variants (c.1172 T > C/ p.Leu391Pro and c.1211G > A/ p.Arg404His) in *FARSA* gene in a Chinese male patient. The protein structure prediction and the analysis of levels of FARSA and FARSB subunits indicated both variants pathogenic. Clinical feature review indicated inflammatory symptoms in young infants may be an additional key feature. Thyroid dysfunction should be considered as a phenotype with variable penetrance.

**Conclusions:**

Our results expanded the current phenotypic and genetic spectrum of FARSA-deficiency.

**Supplementary Information:**

The online version contains supplementary material available at 10.1186/s12920-023-01662-0.

## Introduction

Aminoacyl-tRNA synthetases (ARSs) are indispensable enzymes in the first step of protein biosynthesis by covalently linking a tRNA molecule to its cognate amino acid. ARSs also perform additional functions in key signaling pathways that are not necessarily related to translation. Thirty-seven ARSs have been identified in human, which are classified into two subsets based on their cytoplasmic or mitochondrial localization; 18 function exclusively in the cytoplasm, 17 exclusively in mitochondria, and 2 in both compartments [1, 2]. ARS gene variants are associated with nearly 60 neuropathies, leukoencephalopathies, myopathies, hepatopathies, and lung disorders [3].

The phenylalanyl-tRNA synthetase, FARS1, is located in the cytoplasm and is a hetero-tetrameric structure, consisting of two FARS alpha subunits (FARSA) and two FARS beta subunits (FARSB) [4–6]. Autosomal recessive inheritance of pathogenic variants of *FARSA* or *FARSB* can result in a defective phenylalanyl-tRNA synthetase that causes a multisystemic syndrome (MIM: 619013, 613658) [7]. Patients with defective *FARSA* are characterized by interstitial lung disease, liver disease, brain abnormalities, facial dysmorphism and growth restriction [8–11].

Here, we report a male patient with compound heterozygous missense variants in *FARSA*. We describe the predicted structure and expressional level analysis of the two variant proteins, as well as presenting the patient’s clinical information, including onset, treatment, follow-up and outcome. Our findings indicated that the clinical features of inflammatory symptoms and thyroid dysfunction were different between our patient and the previous reported cases, which might expand the current spectrum of FARSA-deficiency.

## Materials and Methods

### Participant

The proband was a 1-year-old boy. Informed consent was obtained from all the participants and legal guardians for the study. All human subject research reported in this study was in accordance with current ethical standards and was approved by the Institutional Review Board of Beijing Children's Hospital, Capital Medical University (Ethics Approval Number 2015–26).

### Exome sequencing, variant prioritization, and Sanger sequencing

Genomic DNA was extracted from the peripheral blood of the proband and his parents using the TIANamp Blood DNA Kit [Tiangen Biotech (Beijing) Co., Ltd]. Exonic regions were enriched with the Agilent SureSelect Human All Exome V6 Kit (Agilent Technologies, USA) according to manufacturer's instructions and a sequencing library was prepared. High-throughput sequencing was conducted with 150-bp paired-end runs on an Illumina NovaSeq 6000 sequencer (Illumina, USA). The exome sequencing resulted in more than 12 GB of clean data. The average sequencing depth was over 100 × . Sequence alignment to the GRCh37/hg19 human reference genome was conducted according using Burrows-Wheeler Aligner (BWA) and Picard was used to create BAM files. Variant calling was performed using Genome Analysis Toolkit (GATK). Variants were annotated and filtered using Flash Analysis (https://fa.shanyint.com/). The 1000 Genomes Project Database, dbSNP, and gnomAD were used to filter rare variants, and all variants were either absent or present with a minor allele frequency of less than 1 in 1000. The Human Gene Mutation Database, ClinVar, OMIM, and MalaCards databases were used to evaluate the pathogenicity of variants. Multiple prediction algorithms, such as Polyphen-2, SIFT, Mutation taster, and CADD, were used to predict the harmfulness of variants. The pathogenicity of variants was classified according to the standards and guidelines of the American College of Medical Genetics and Genomics (ACMG) [12]. Sanger sequencing was performed using an ABI 3730xl DNA Analyzer (Applied Biosystems, USA) and primers were designed to amplify the regions encompassing the variants.

### Peripheral blood mononuclear cell (PBMC) isolation

Fresh peripheral blood was collected and an equal volume of phosphate buffered saline (PBS) added. Two diluted blood volumes were carefully laid over one volume of Ficoll (Tbdscience, China) by resting the pipette tip against the tube wall. The samples were then centrifuged at 1,000 × g at room temperature for 30 min, which separated the samples into the following layers: plasma, PBMCs, Ficoll, and red blood cells (RBCs). The PBMC layer was carefully collected and transferred to a fresh tube. The PBMCs were washed with PBS and centrifuged at 700 × g for 5 min at room temperature. The supernatant was discarded and the PBMCs lysed with lysis buffer (50 mM Tris, 150 mM NaCl, 1% Triton X-100, and pH 7.4) containing protease inhibitor (Sigma, USA). The lysate was then subjected to sodium dodecyl sulfate–polyacrylamide gel electrophoresis (SDS-PAGE).

### Western blot

SDS-PAGE was performed using 4%–15% gradient gels (Applygen Technologies, China). Proteins were electrophoretically transferred to polyvinylidene fluoride (PVDF) membranes. Membranes were then blocked with 5% skimmed milk, and incubated with antibodies. Anti-β-Actin, FARSA, and FARSB antibodies were purchased from Merck (Germany), Novus (USA), and Santa Cruz (USA), respectively. Image J was used for analysis of detected protein bands. The relative protein levels were calculated using FARSA/β-Actin and FARSB/β-Actin ratios.

### Structure modeling

The crystal structure of human FARS1 (PDB: 3L4G) [6] was aligned with the structure of *Thermus thermophiles* tRNA^Phe^ molecule (PDB: 2IY5) [13] using UCSF chimera [14] to model the position of tRNA^Phe^. The Leu391Pro and Arg404His mutations were created using Phyre 2 [15] with human FARSA as a template. The structures were rendered and analyzed using UCSF chimera. Dynamut2 was used to predict the impact of the mutations on protein stability [16].

## Results

### Clinical information

The male proband was born to healthy gravida 1 parity 1 (G1P1) mother and a father who were non-consanguineous. The boy could say "mama" at 6 months old; however, after the age of 8 months he could no longer say “mama” and could just say "a ~ ". At the age of one year, he could raise his head, roll over and sit all by himself.

He was referred to the local hospital at 7 months old because of jaundice of unknown origin. The condition improved after taking cephalosporin and the traditional Chinese medicine, Yinzhihuang, for one month. At the age of 1 year, he was referred to the hospital because of a fever and cough. Routine blood examination showed increased white blood cell (WBC: 16.71 × 10^9/L) and neutrophil (NEUT: 11.71 × 10^9/L) numbers. His blood biochemical index indicated a decreased level of serum albumin. Serum alanine aminotransferase (121.3–158.5U/L) and aspartate aminotransferase (294.5–588.0U/L) levels were increased. Levels of total bilirubin (TBIL: 32.33umol/L) and direct bilirubin (DBIL: 24.54umol/L), alpha-fetoprotein (AFP > 1000 IU/ml), carcinoembryonic antigen (CEA6.34 ng/ml) and anti-thyroglobulin antibodies (33.75 IU/ml) were all elevated. Free triiodothyronine (1.18 pg/ml) and free thyroxine (0.51 ng/dl) levels were decreased. These results indicate liver involvement and thyroid dysfunction. Cytomegalovirus IgM antibody was positive. Chest CT showed bilateral subpleural ground glass shadows in the lungs. Brain magnetic resonance imaging was nonspecific. Echocardiography revealed thickening of the endocardium of the left ventricle. The condition of fever and cough improved after taking ganciclovir and cephalosporin for two weeks. However, the white blood cell count (WBC: 17 × 10^9/L) was still abnormally elevated after taking cephalosporin for one month.

At the age of 13 months, the patient was referred to Beijing Children’s Hospital and assigned to multi-disciplinary treatment. A repeated routine blood examination (WBC: 29.63 × 10^9/L; NEUT: 15.19 × 10^9/L) and biochemical index (ALT: 63.8U/L; AST: 182.1U/L; TBIL: 32.77umol/L; DBIL: 20.21umol/L; IBIL: 12.56umol/L) indicated that the abnormalities listed above were still present. Abdominal ultrasonography showed diffuse enhancement of liver parenchyma echo, a hypo-fat area beside the gallbladder, and low echo in the left lobe of the liver. Color Doppler echocardiography revealed a small pericardial effusion. There were no obvious abnormalities in left and right cardiac functions.

**Fig.1:**
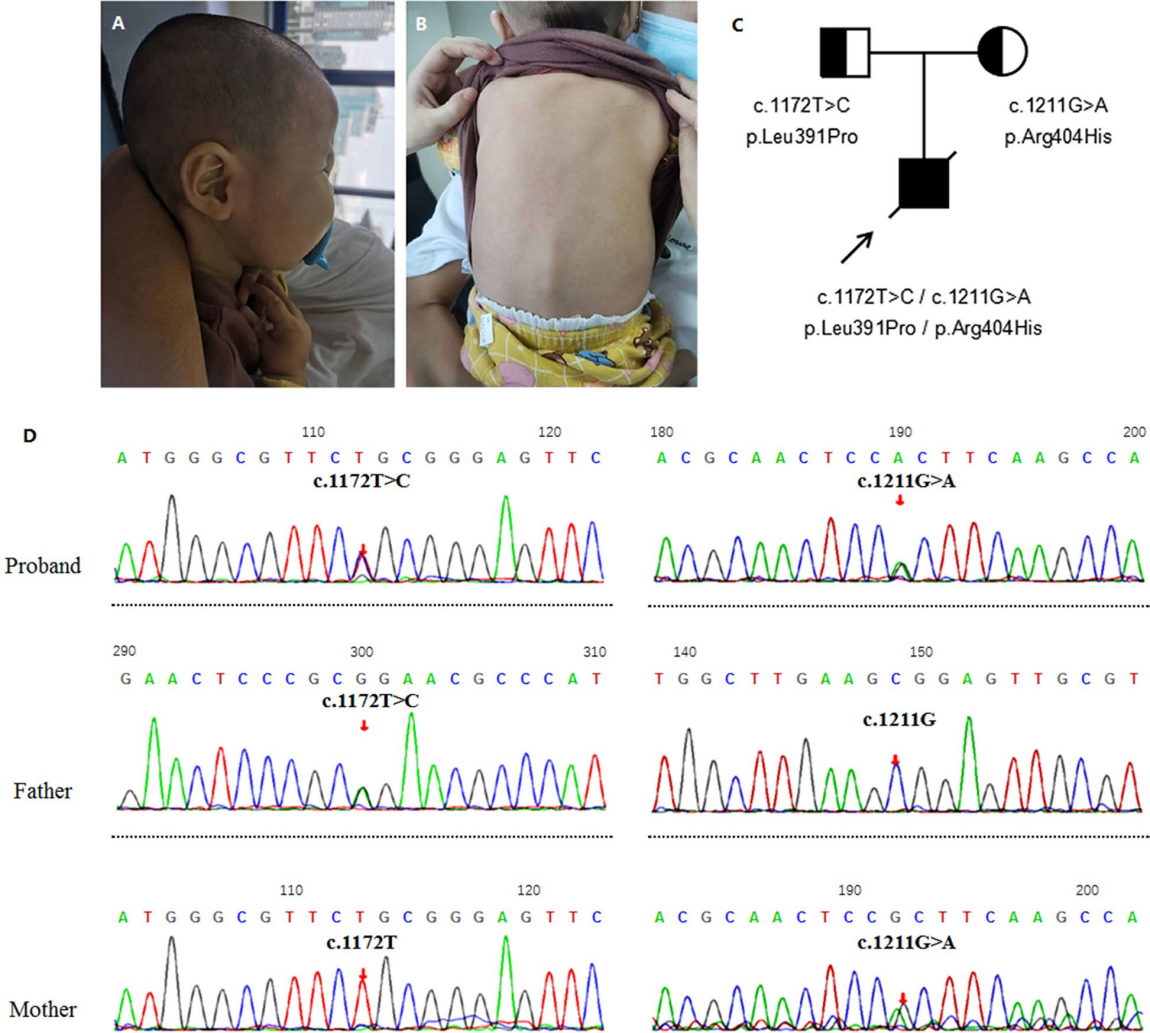
The view, pedigree, and Sanger sequencing of the patient. **A** and **B** The side view and the back view of the male patient. **C** and **D** The pedigree and Sanger sequencing of the proband and his parents

The boy's height was 75 cm (<P_25_). He weighed 6.95 kg (<P_1_) and had a head circumference of 43 cm (<P_3_). His anterior fontanelle was flat and soft. He had a Cushing syndrome-like face, with deep-set eyes and raised eyebrow arch. He had poor muscle strength and hypotonia (Fig. 1A-B).

### Follow-up and clinical outcome

After a multi-disciplinary treatment consultation, the proband returned to the local hospital for subsequent treatment. A follow-up study was conducted for 3 months. During this period, the patient had a cough, diarrhea three times a day, and a fever up to 38.5°C. A routine blood test showed elevated white blood cell count and C-reactive protein (CRP) level. Cefixime was administered orally (to protect his liver) for 3–4 days and returned the body temperature to normal and his cough and diarrhea lasted for a week. Thereafter, the patient was given Peptamen Junior and ate a normal diet. The patient was conscious and his appetite, sleep, and urination were normal.

Monthly abdominal ultrasound showed the proband’s liver to be light and large with diffuse injury and with hypoechoic nodules in the left lobe, and with bile wall edema and a large amount of ascites. Serum biochemistry showed reduced total protein and albumin levels, and significantly elevated levels of aspartate aminotransferase and γ-glutamyltranspeptidas. A high number of white blood cells and neutrophils were detected. No treatment was given. The patient died of dyspnea a month later.

### Genetic findings

**Fig. 2:**
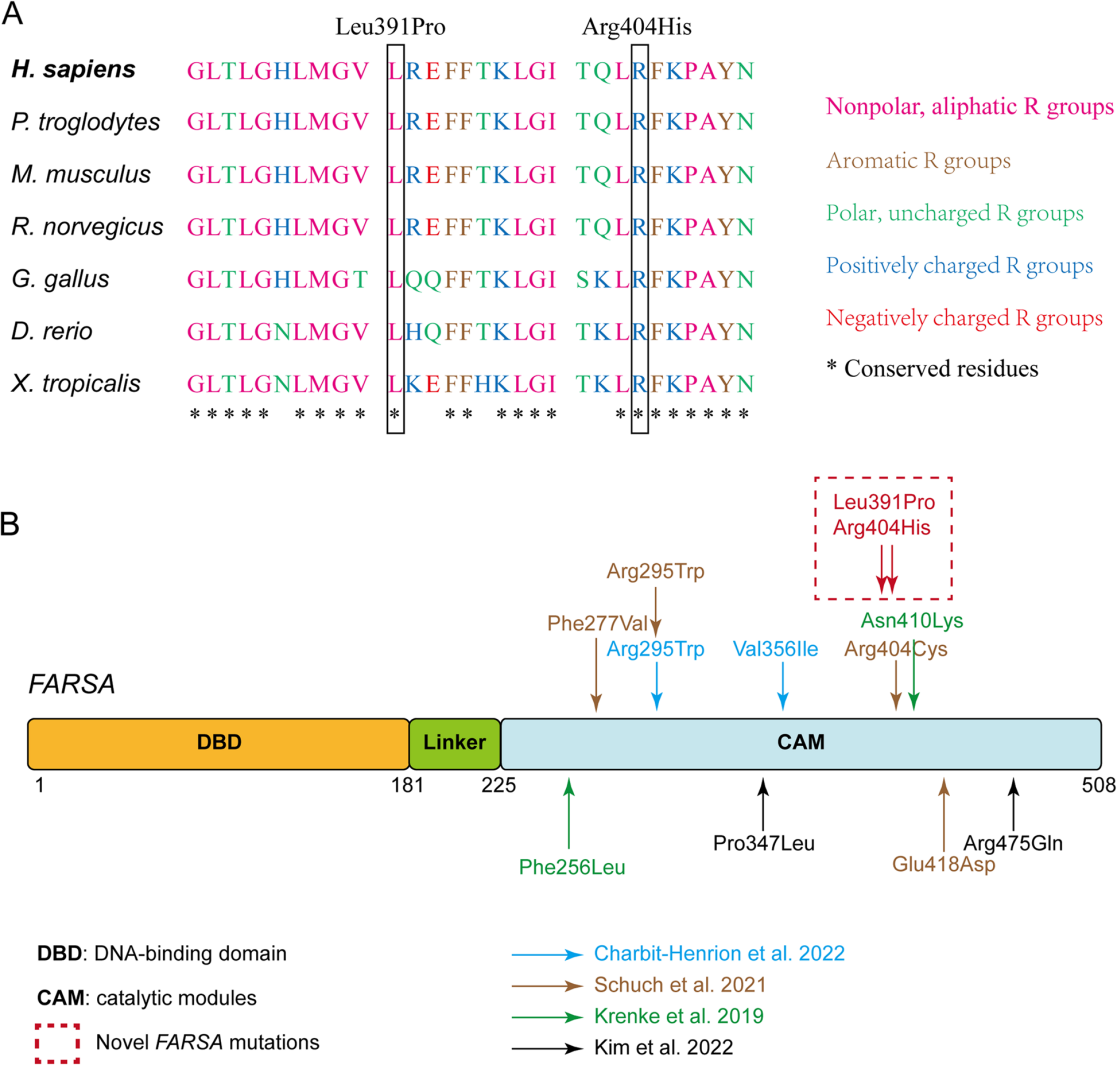
Sequence alignment of the variants and reported cases. **A** Sequence alignment indicates the location and conservation status among orthologs of FARSA variants. **B** Schematic of FARSA protein topology with location of missense mutations in the CAM domain

We performed exome sequencing of DNA isolated from the blood of the proband and his unaffected parents. We identified two candidate variants of unknown significance in the *FARSA* gene that met the inheritance pattern and allele frequency criteria, a paternal missense variant, c.1172 T > C (p.Leu391Pro), and a maternal missense variant, c.1211G > A (p.Arg404His). Sanger sequencing showed that the proband's father was heterozygous for c.1172 T > C and his mother was heterozygous for c.1211G > A (Fig. 1C and D). The variant c.1172 T > C is located in exon 10 while c.1172 T > C is located in exon 11 of *FARSA* (NM_004461). Sequence alignment indicated that the two variants were conserved among most species (Fig. 2A). Both variants are rare in the normal population. c.1172 T > C was not recorded in the 1000 Genomes Project Database, dbSNP, or gnomAD database. c.1211G > A was recorded in the gnomAD database with a very low allele frequency in a European population (0.00001759, 2/113,722). (Evidence of PM2, according to ACMG guideline) [12]. We used multiple in silico algorithms, including SIFT, Polyphen-2, CADD, MutationTaster, and M-CAP, to predict the effects of the missense variants. Both variants were predicted to be either damaging or probably pathogenic (Supplemental Table 1) (Evidence of PP3). No other rare variant, which correlated highly with clinical phenotypes and met the inheritance pattern, was identified in the exome sequencing data.

### Clinical features review and protein structure prediction

Only 9 cases caused by *FARSA* deficiency have been described worldwide (Table 1). A review of the clinical features of the nine unrelated patients reveals a complex phenotype with interstitial lung disease (ILD), brain abnormalities, growth delay, and hepatomegaly. More than half of the patients had respiratory and hepatic abnormalities, neurodevelopmental delay, failure to thrive, and hypoalbuminemia. In our patient, hypothyroidism and obvious microcephaly (head circumference: 43 cm at the age of 13 months, < P_3_) with no abnormalities on brain magnetic resonance imaging are less frequent findings among other reported patients carrying *FARSA* mutations. The primary manifestation is a progressive deterioration of liver function during early childhood, which may reveal the heterogeneity of clinical features in our patient.

**Table 1:** Summarized genotype and clinical phenotype of patients with mutations in *FARSA* gene

Genotype and Clinical phenotype		FARSA subjects										
		1	2	3			4				5	Σ
		P1	P2	P3	P4	P5	P6	P7	P8	P9	P10	
Genotype of *FARSA* gene (variant 1, variant 2)		L391P R404H	F256L N410K	R404C E418D	R295W R295W	F277V F277V	R295W R295W		V356I V356I		R475Q P347L	-
Respiratory system	interstitial lung disease	•	•	•	•	•	•		•	•	•	9/10
	cholesterol pneumonitis		•	•	•	•	•		•			6/10
	pulmonary alveolar proteinosis				•		•		•			3/10
	cystic lung disease		•	•		•						3/10
	digital clubbing		•	•		•	•	•				5/10
Growth	failure to thrive	•	•	•	•	•	•	•	•	•	•	10/10
	feeding difficulty/diarrhea	•	•		•		•	•			•	6/10
Liver	hepatomegaly/ hepatosplenomegaly	•	•	•	•		•		•	•	•	8/10
	abnormal liver values (blood)	•	•	•	•	•					•	6/10
	liver steatosis/ hyperechogenicity		•	•	•		•		•		•	6/10
Musculature	hypotonia	•	•	•	•						•	5/10
	decreased muscle mass		•			•						2/10
	abnormal muscle histology			•								1/10
Nervous system	neurodevelopmental/ speech delay	•		•	•				•	•	•	6/10
	headache			•								1/10
	microcephaly	•							•	•		3/10
	brain cysts		•			•						2/10
	brain calcifications		•								•	2/10
	white matter lesions			•			•		•	•		4/10
	brain aneurysm			•						•		2/10
Skeletal system	pectus carinatum/ excavatum		•	•								2/10
	joint hyperflexibility		•	•								2/10
	osteopenia			•	•							2/10
	Marfan-like syndrome		•	•								2/10
Endocrine system	growth hormone resistance/ deficiency			•		•						2/10
	hypothyroidism	•	•								•	3/10
Dysmorphic features	face/body appearance	•	•	•								3/10
Eye	abnormal eye movement				•							1/10
Ear	sensorineural hearing loss					•						1/10
Cardiovascular system	structural heart/vessel defects	•		•		•						3/10
Immune system	abnormal blood cell counts	•	•								•	3/10
	IgG deficiency				•							1/10
	hypoalbuminemia	•	•	•	•	•	•	•	•	•		9/10
	Chronic inflammation	•					•	•	•	•		5/10
Gastrointestinal tract	hernia		•									1/10
Urinary system	vesicoureteral reflux		•									1/10
	nephrolithiasis					•						1/10
	hyperphosphaturia				•							1/10
	tubulopathy				•						•	2/10
	proteinuria										•	1/10
Skin	poor wound healing			•								1/10
	abnormal subcutaneous fat tissue		•		•							2/10

1 (Patient 1, P1), presented here; 2, (Patient 2, P2), Krenke. et al. presented; 3 (Patient 3–5, P3-P5), Schuch et al. presented; 4 (Patient 6–9, P6-P9), Charbit-Henrion et al. Presented; 5 (Patient 10, P10), Kin et al. presented. The dark spot reflects positivity, empty reflects negativity or unknown status

**Fig. 3:**
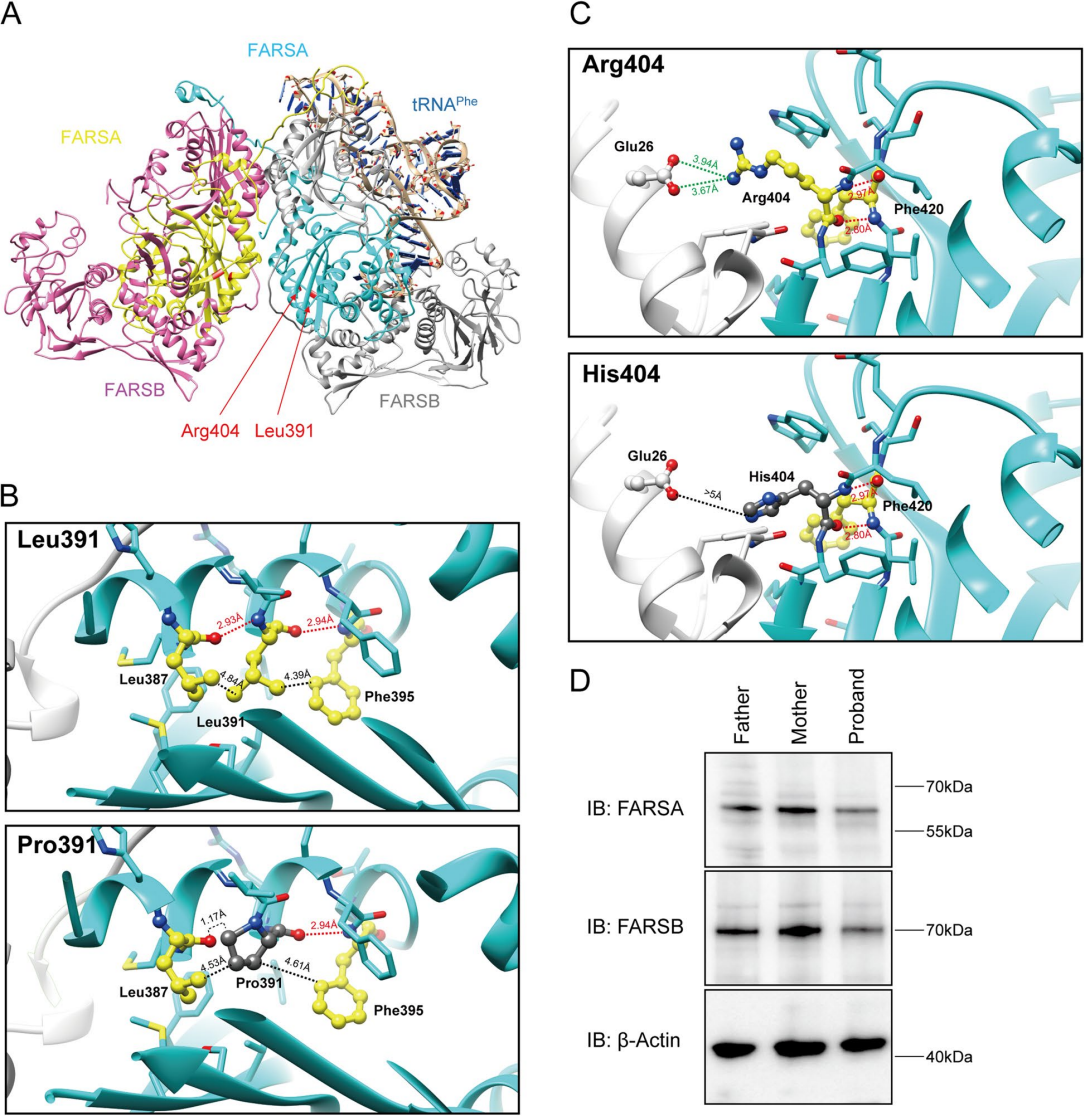
Predicted impact of mutations on FARSA protein structure and the protein expression levels of FARSA and FARSB in the proband and his parents. **A** Location of mutations in the structural model of FARS1complexed with tRNA^Phe^ in cartoon backbone representation. Two FARSA chains are colored in cyan and yellow, two FARSB chains in dark gray and pink, tRNA^Phe^ in blue. Mutated amino acid residues are shown in red. **B** and **C** Predicted impact of mutations on local amino acid interactions with pairwise comparison between side chains of wild-type residues and modeled missense mutations (B, Leu391 and Pro391; C, Arg404 and His404). The residues Leu391 and Arg404 are colored yellow and showed as ball and stick model. They formed hydrogen bond with Leu387, Phe395, and Phe420 residues are also colored yellow and drawn ball and stick. Mutated residues Pro391 and His404 are shown in dim gray. FARSA chain is colored cyan and FARSB chain is colored light gray. Blue and red balls highlight single nitrogen and oxygen, respectively. **D** FARSA and FARSB expression levels in the proband and his parents

*FARSA* encodes the 508-amino acid α-subunit of the PheRS complex. The crystal structure reveals that FARS comprises an N-terminal tRNA-binding domain (DBD) followed by the linker region, and the catalytic module (CAM). All variants identified were located exclusively in the CAM domain (Fig. 2B). By forming a heterotetramer with the FARSB subunit, FARSA conjugates phenylalanine to the cognate tRNA. To investigate the impact of the variants on FARS1 structure and function, we aligned *T. thermophilus* tRNA^Phe^ (PDB: 2IY5) with the crystal structure of human FARS1 (PDB: 3L4G) using UCSF chimera. In this model, Leu391 and Arg404 are located near the phenylalanine binding pocket (Fig. 3A). Conformational analysis suggested that Leu391 formed two hydrogen bonds with Leu387 (2.93 Å) and Phe395 (2.94 Å). The substitution by Proline at this position will only form one hydrogen bond (red dotted line) with Phe395 (2.94 Å), but very likely clash with Leu387 (1.17 Å) in protein conformation. The spaces of residue 391 and Leu387 become more crowded (black dotted line, distance from 4.84 Å to 4.53 Å) (Fig. 3B). The other variants Arg404His primarily affect residues close to interfaces between subunits A/B. Arg404 can form an ionic bond (green dotted line) with Glu26 (3.94 Å and 3.67 Å) in the FARSB subunit. A substitution by Histidine would impact the ionic bond network (distance with Glu26 > 5 Å) and might interfere with the overall structure of the FARS1 complex (Fig. 3C). We also remodeled FARSA and FARSB proteins using another protein fold recognition server Phyre2 (http://www.sbg.bio.ic.ac.uk/phyre2) and checked for spaces. The results showed that mutations will affect the distance with around residues (Supplemental Fig. 1A). According to the structure information, the prediction of protein stability changes upon mutation scored Leu-to-Pro391 and Arg-to-His404 as energetically destabilizing (ΔΔG = -0.35 kcal/mol; ΔΔG = -0.7 kcal/mol, respectively) using the Dynamut2 tool, overall indicating that those variants could affect FARSA protein stability.

### Levels of FARSA and FARSB Subunits

To further characterize the effect of the compound heterozygous missense variants, we compared the levels of FARS α-subunit (FARSA) and β-subunit (FARSB) in the peripheral blood of the proband and his parents. The average protein expression levels of his parents were used as normal control. The level of FARSA was 35% lower and that of FARSLB was 39% lower in the proband compared with the respective average values of his parents (Fig. 3D and Supplemental Fig. 1B, C). These results indicate that the α-chain and β-chain require each other for stabilization.

## Discussion

*FARSA* deficiency can result in FARS1-related disorder, which is characterized by interstitial lung disease, growth delay, hypotonia, brain calcifications, and liver dysfunction. To date, nine patients with *FARSA* deficiency from seven unrelated families have been reported [8–11]. Our study describes an additional patient with two missense variants that enlarges both the genetic and the phenotypic spectrum of *FARSA* deficiency. The patient shares some typical symptoms of other *FARSA*-deficiency cases, including lung disease, microcephaly, hypotonia, liver involvement, and growth retardation. Some previously reported patients with *FARSA*-deficiency display intracranial calcifications on cranial magnetic resonance imaging (MRI). Our patient presented obvious microcephaly (< P_3_) and developmental delay, but no abnormalities on brain magnetic resonance imaging.

Among the published cases, four of nine patients displayed autoimmunity with chronically elevated CRP and leukocytosis without any evidence of infection. In our case, the patient presented a long-term elevated white blood cell count and increased levels of CRP, and medication did not relieve these measures. These inflammatory markers in young infants may be a key feature of *FARSA*-deficiency.

Four patients reported [8, 9, 11] had endocrinology involvement, including hypopituitarism, hypothyroidism, growth hormone resistance, or growth hormone deficiency; however, five other cases did not present these features. Our patient displayed thyroid dysfunction, with elevated anti-thyroglobulin antibodies and decreased levels of free triiodothyronine. This clinical information indicates that thyroid dysfunction should be considered as a phenotype with variable penetrance.

According to the Human Gene Mutation Database (Professional v.2022.4), seven missense *FARSA* variants (F256L, F277V, R295W, V356I, R404C, N410K, and E418D) [8–10] have been identified as pathogenic and to result in FARSA deficiency. All seven variants were located in the phenylalanine-binding pocket domain of *FARSA*. In our case, the patient carried two heterozygous missense variants, p.R404H and p.L391P. P.L391P is also located in the phenylalanine-binding pocket domain but was not present in published databases. R404C was previously reported in a girl with *FARSA* deficiency [10], and functional assessment of R404 suggests that this variant would reduce the activity of the FARS1 enzyme (Evidence of PM5 according to ACMG guideline) [12]. In a structural model of the FARS1 complex, R404 is predicted to impact interactions between the FARSA and FARSB subunits. The two variants in our case were rare in the normal population and in silico prediction indicated the two variants to have damaging effects (Evidence of PM2 and PP3). The reduced levels of FARSA and FARSB in the patient strongly indicated that the two variants have a noticeable effect on the level of the FARS1 complex (Evidence of PS3), which were the new evidence for the variants’ pathogenicity classification According to the 2015 American College of Medical Genetics and Genomics (ACMG) and Association for Molecular Pathology (AMP) guidelines [12], p.R404H (PS3 + PM2 + PM5 + PP3) and p.L391P (PS3 + PM2 + PP3) can be classified as likely pathogenic, resulting in FARSA deficiency disorder.

The molecular structure of PheRS is a tetramer including both FARSA and FARSB proteins. Recently, a series of findings indicate that a disease similar to a syndrome associated with FARSB defects can also be caused by FARSA mutations. Our patient comprising hepatomegaly, hypoalbuminemia, and elevated aminotransferase were also consistent with the FARSB disease. But hypothyroidism was a rare symptom that was only found in two patients with FARSA deficiency reported by Krenke. et al. and Kim. et al. This observation indicates that patients with hypothyroidism may not be ignored and ruled out of FARS1 defect.

ARS1-related disease with autosomal recessive inheritance often causes severe clinical phenotypes involving many organs and with onset in the first year of life. Patients with ARS1-related disease usually have exacerbated symptoms during infectious episodes that may lead to premature death; however, there is currently no effective treatment strategy for ARS-deficient patients. For our patient, their first visit to a hospital was 6–7 months after birth. The main manifestation was progressive deterioration of liver function. Routine treatment to protect the liver was ineffective. The child developed hypoproteinemia, massive ascites, and finally died because of dyspnea at approximately 2 years old. This lifetime was shorter than that of other FARSA-deficiency cases. Before the definite molecular diagnosis, the patient received treatment that lacked specificity and was ineffective, which might be the reason for the early death.

In conclusion, in addition to the common key phenotypic features of FARSA-deficiency, inflammatory symptoms in young infants may be an additional key feature. Thyroid dysfunction should be considered as a phenotype with variable penetrance. Moreover, our findings indicate that molecular genetic diagnosis should be considered as a first tier test for FARS1-related disease patients to guide personalized clinical management. In addition, our results expand the current phenotypic and genetic spectrum of *FARSA*-associated recessive disease.

### Supplementary Information

**Additional file 1.** **Supplemental Table 1.** Genomic information and *in silico* prediction of the effects of the two variants.

**Additional file 2.**

## Data Availability

The raw sequence data that support the findings of this study have been deposited in the Genome Sequence Archive [17] in National Genomics Data Center [18], China National Center for Bioinformation / Beijing Institute of Genomics, Chinese Academy of Sciences (GSA-Human: HRA005459) that are publicly accessible at https://ngdc.cncb.ac.cn/gsa-human.
