# Supplementary material for: Phenylalanyl-tRNA synthetase deficiency caused by biallelic variants in FARSA gene and literature review
Source: BMC Med Genomics. 2023 Oct 13;16:245. doi: 10.1186/s12920-023-01662-0 (PMC10571242; doi:10.1186/s12920-023-01662-0)
Supplement: Supplementary file 1 — Additional file 1. Supplemental Table 1. Genomic information and in silico prediction of the effects of the two variants. [file 12920_2023_1662_MOESM1_ESM.docx]

Supplemental Table 1. Genomic information and *in silico* prediction of the effects of the two variants.

|  | ***FARSA*: c.1172T>C** | ***FARSA*: c.1211G>A** |
| --- | --- | --- |
| **Chromosome location** | chr19:13035476 (hg19) | chr19:13035325 (hg19) |
| **Amino acid change** | p.Leu391Pro | p.Arg404His |
| ***In silico* prediction** |  |  |
| SIFT | Damaging (score: 0.001) | Damaging (score: 0.001) |
| Polyphen-2_HDIV | Probably damaging (score: 1.0) | Probably damaging (score: 1.0) |
| Polyphen-2_HVAR | Probably damaging (score: 1.0) | Probably damaging (score: 0.966) |
| MutationTaster | Disease causing (score: 1.0) | Disease causing (score: 1.0) |
| M-CAP | Damaging (score: 0.337) | Damaging (score: 0.352) |
| CADD | Damaging (score: 25.1) | Damaging (score: 35) |
