# Supplementary material for: Phenylalanyl-tRNA synthetase deficiency caused by biallelic variants in FARSA gene and literature review
Source: BMC Med Genomics. 2023 Oct 13;16:245. doi: 10.1186/s12920-023-01662-0 (PMC10571242; doi:10.1186/s12920-023-01662-0)
Supplement: Supplementary file 2 — Additional file 2. [file 12920_2023_1662_MOESM2_ESM.pptx]

## Slide 1
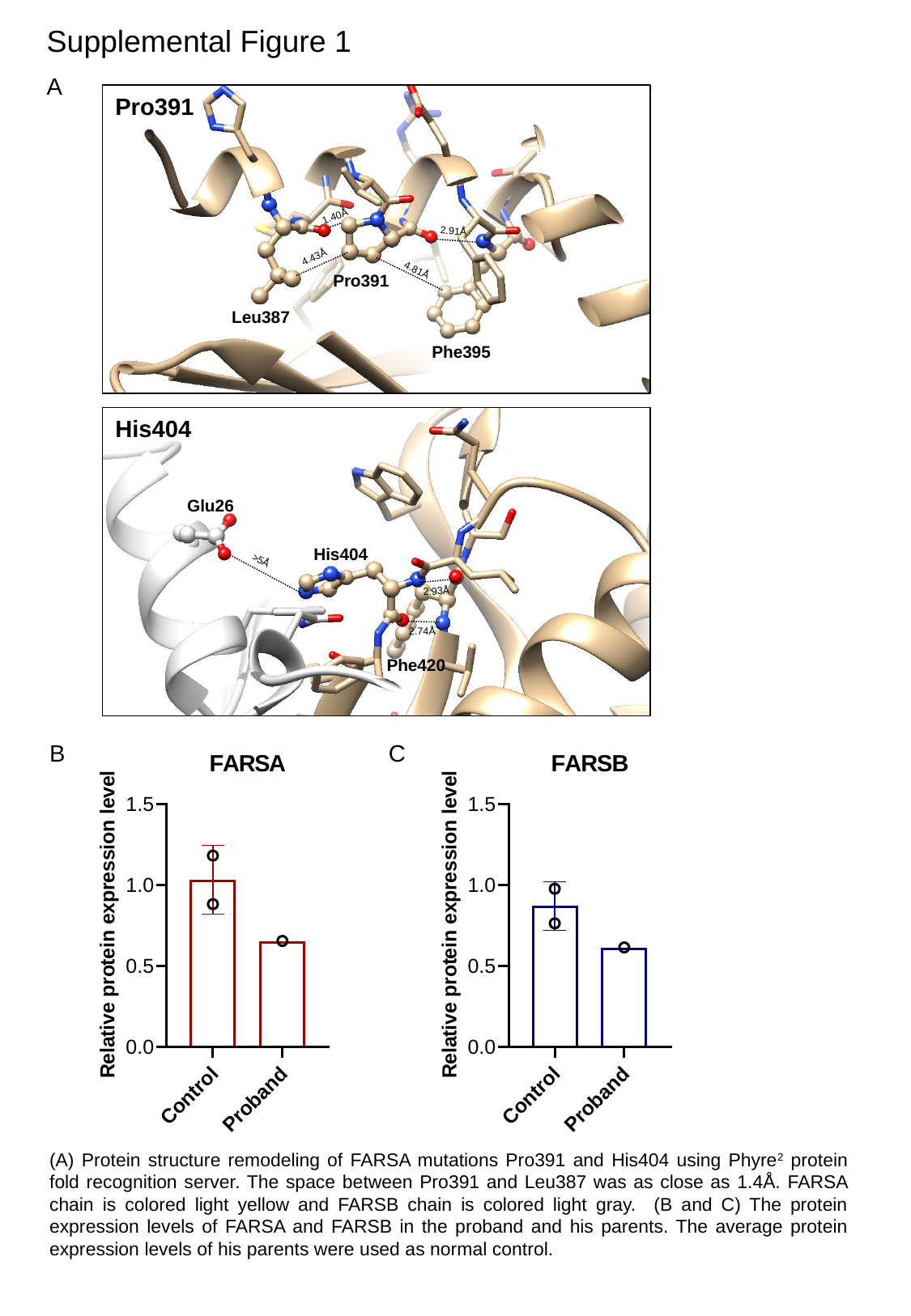

Supplemental Figure 1
A
Pro391
1.40Å
2.91Å
4.43Å
4.81Å
Pro391
Leu387
Phe395
His404
Glu26
His404
>5Å
2.93Å
2.74Å
Phe420
B
C
(A) Protein structure remodeling of FARSA mutations Pro391 and His404 using Phyre2 protein fold recognition server. The space between Pro391 and Leu387 was as close as 1.4Å. FARSA chain is colored light yellow and FARSB chain is colored light gray. (B and C) The protein expression levels of FARSA and FARSB in the proband and his parents. The average protein expression levels of his parents were used as normal control.

## Slide 2
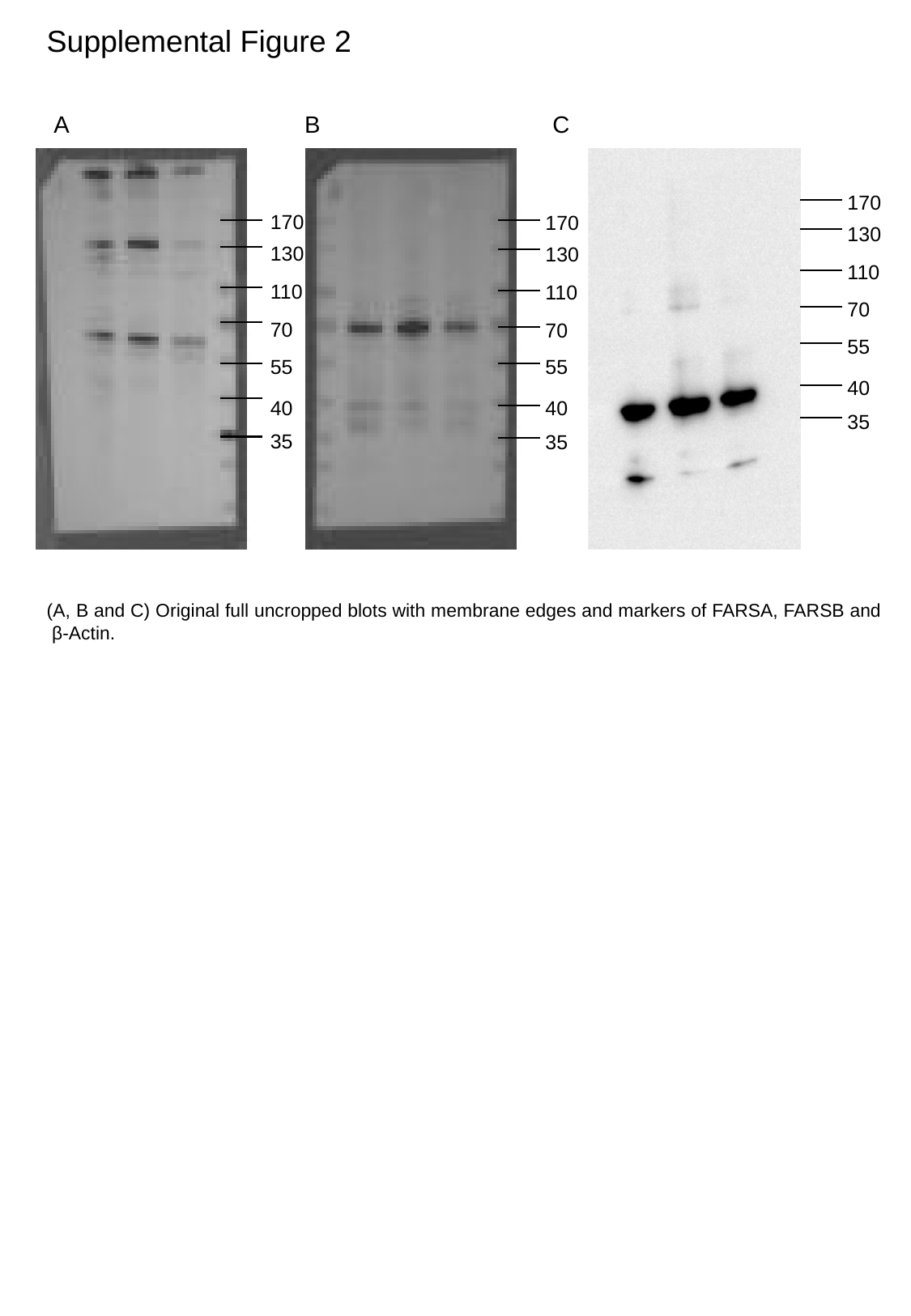

Supplemental Figure 2
A
B
C
170
170
170
130
130
130
110
110
110
70
70
70
55
55
55
40
40
40
35
35
35
(A, B and C) Original full uncropped blots with membrane edges and markers of FARSA, FARSB and β-Actin.
